# Responses of nitrobenzene removal performance and microbial community by modified biochar supported zerovalent iron in anaerobic soil

**DOI:** 10.1038/s41598-024-67301-5

**Published:** 2024-07-24

**Authors:** Lu Hainan, Li Peng, Li Qingqing, Liu Fang, Zhou Dong, Huang Shenfa, Yang Jie, Li Zhiheng

**Affiliations:** 1 Ministry of Ecology and Environment Engineering Center for Urban Soil Contamination Control and Remediation, Shanghai Academy of Environmental Sciences, Shanghai, 200233 China; 2 College of Environmental Science and Engineering, Donghua University, Shanghai, 201620 China; 3 Shanghai Technology Center for Reduction of Pollution and Carbon Emissions, Shanghai, 200235 China; 4 School of Environmental Science and Engineering, Key Laboratory of Solid Waste Treatment and Recycling of Zhejiang Province, Zhejiang Gongshang University, Hangzhou, 310018 Zhejiang Province China

**Keywords:** Modified biochar, ZVI, Microbial community, Nitrobenzene, Anaerobic soil, Environmental chemistry, Pollution remediation

## Abstract

Biochar-supported ZVI have received increasing attention for their potential to remove nitrobenzene in groundwater and soil. However, the capacity of this material to enhance the biological reduction of nitrobenzene and alter microbial communities in anaerobic groundwater have not been explored. In this study, the nitrobenzene removal performance and mechanism of modified biochar-supported zerovalent iron (ZVI) composites were explored in anaerobic soil. The results showed that the 700 °C biochar composite enhanced the removal of nitrobenzene and inhibited its release from soil to the aqueous phase. NaOH-700-Fe50 had the highest removal rate of nitrobenzene, reaching 64.4%. However, the 300 °C biochar composite inhibited the removal of nitrobenzene. Microbial degradation rather than ZVI-mediated reduction was the main nitrobenzene removal pathway. The biochar composites changed the richness and diversity of microbial communities. ZVI enhanced the symbiotic relationship between microbial genera and weakened competition between soil microbial genera. In summary, the 700 °C modified biochar composite enhanced the removal of nitrobenzene by increasing microbial community richness and diversity, by upregulating functional genes, and by promoting electron transfer. Overall, the modified biochar-supported ZVI composites could be used for soil remediation, and NaOH-700-Fe50 is a promising composite material for the on-site remediation of nitrobenzene-contaminated groundwater.

## Introduction

Nitrobenzene, a typical nitroaromatic compound, is widely used in industries such as pharmaceutical production, dye production, and pesticide production^1^. However, large amounts of wastewater containing nitrobenzene are generated during its production and use, leading to soil and groundwater pollution if not handled properly. As a result, nitrobenzene has been listed as a priority contaminant in many countries^2^. The U.S. Environmental Protection Agency (USEPA) found that nearly 3% of wastewater discharged by factories contained over 100 mg/L nitrobenzene^3^. Therefore, it is necessary to remediate nitrobenzene-containing soil and groundwater in contaminated sites.

Many physical, chemical, and biological methods, such as chemical oxidation, chemical reduction, biodegradation, and sorption^4–8^, have been successfully used to remove nitrobenzene. Among these methods, reduction is more efficient at transforming nitrobenzene into less toxic aniline. Zerovalent iron (ZVI), which has strong reducing capacity and large specific surface area, is widely used for removing contaminants, such as chlorinated organic pollutants, nitroaromatic compounds, and heavy metals, in different media^9–11^, and ZVI has been applied in the field for pollutant remediation^12,13^. However, there are still obvious obstacles, such as issues related to passivation, aggregation, and difficulties in migration, that need to be overcome before large-scale application is possible. Currently, modification, such as noble metal doping, sulfidation, and polymer loading, is a good method for enhancing the performance of ZVI^14–16^. These modifications can improve the reactivity, stability, and injectability of ZVI to improve the removal efficiency of organic pollutants^17–19^.

Biochar is a carbon-rich material that can be produced through thermal decomposition of biomass under conditions of limited or no oxygen at specific temperature (< 900 °C)^20,21^. Biochar has a large specific surface area, abundant surface functional groups, and strong electron transfer ability. Its porous structure enables the even dispersion of ZVI on its surface and in its pores via sorption, coordination, chelation, etc., which can slow the aggregation and oxidation of ZVI and improve its reactivity with target pollutants^22^. For example, the aggregation of nano zero-valent iron (nZVI) can be effectively prevented by uniformly distributing nZVI particles throughout entire carbon microspheres, which has been shown to lead to enhanced trichloroethylene removal^23^. Additionally, Wang et al. synthesized biochar-loaded nZVI composite materials for the degradation of nitrophenol and found that biochar prevented the aggregation of nZVI and promoted the degradation of nitrophenol in aqueous solution^24^. Moreover, chemical modification can further improve the sorption and loading performance of biochar for ZVI, enhancing its ability to remove organic pollutants^25,26^. The dispersion and thermal stability of nano Pd/ zero-valent iron (nPd/Fes) were enhanced by alkali modification^27^. Although the removal performance, removal mechanism, and electron transfer process of organic pollutants in the presence of biochar-supported ZVI materials in aqueous systems have been well explored^28–30^, there are limited reports on the effectiveness of these materials for the removal of nitrobenzene in groundwater. Due to its low solubility, nitrobenzene is difficult to dissolve into the aqueous phase and can persist for a long time as groundwater contaminates aquifers^31^. In addition, nitrobenzene has high hydrophobicity and is easily adsorbed by soil particles, leading to its accumulation in the soil phase rather than in the aqueous phase. Pollutants in the soil can be adsorbed onto biochar-supported ZVI materials; this process affects the pollutant removal efficiency through adsorption, distribution, and reduction. During remediation, the rate of ZVI-mediated reduction reactions gradually decreases, which will affect the removal efficiency and pathways of organic pollutants and may affect microbial community structure, which is another important factor in organic pollutant removal. Thus, it is meaningful to explore the remediation of nitrobenzene in soil and groundwater to investigate the underlying mechanism of nitrobenzene removal by modified biochar-supported ZVI.

In organic compound-contaminated soil and groundwater, biodegradation is an important contributor to pollutant remediation. The porous structure of biochar can provide attachment sites for microorganisms and binding sites for toxic compounds^32^. ZVI-mediated anaerobic systems are used for the reduction and transformation of various pollutants and can create anaerobic conditions for microbial degradation by reducing the oxidation‒reduction potential (ORP) and acidity^33,34^. For example, biochar can improve the biological reduction of nitrobenzene by *Shewanella oneidensis* MR-1 in anaerobic systems^35^. Moreover, biochar can stabilize S-nZVI (S-nZVI@BC), and the biological anaerobic reduction performance of nitrobenzene was improved compared to that of nZVI^36^. However, these studies were conducted in controlled anaerobic reactors seeded with anaerobic sludge or in pure culture microbial systems. The feasibility of using modified biochar-supported ZVI to enhance the biological reduction of nitrobenzene and the impact of this material on the removal of nitrobenzene and changes in the microbial community in anaerobic groundwater and soil have yet to be explored.

Compared to the chemical solvent method, which needed excess chemical solution and generated a large amount of wastewater^37^, ball milling is a well-known mechanochemical technology, which has advantages of green, low cost, simple operation and mass production as an emerging technology applied to the preparation of composites^38,39^. Ball milling process could increase the specific surface area and pore structure of biochar, and provide more active adsorption sites, which could accelerate removal of organic pollutants^40,41^. In addition, ball milling process could enhance the dispersibility of iron oxide on biochar surfaces, in which the shear force could break the iron oxide layer in order to expose new active sites on ZVI surface^42–44^.The purpose of this study was to investigate the nitrobenzene removal performance of modified biochar-supported ZVI materials in an anaerobic soil environment. The effectiveness of the composites in removing nitrobenzene under anaerobic soil conditions was also analyzed. The influence of different Fe/biochar ratios and modification agents was studied. The impact of microbial communities during the experiment was also investigated. This research provides new insights for the application of modified biochar-supported ZVI composite materials for on-site removal of nitrobenzene from aquifers.

## Materials and methods

### Materials

Nitric acid (HNO_3_), hydrochloric acid (HCl), and sodium hydroxide (NaOH) were purchased from Sinopharm Chemical Reagent Co., Ltd. Nitrobenzene (99%), ZVI (metal basis grade, 99.9%, 100 mesh) and methanol (HPLC grade) were purchased from Aladdin Chemical Reagent Co., Ltd.

### Preparation of modified biochar

Wheat straw was collected, oven-dried overnight at 80 °C, crushed and sieved to < 0.355 mm. The biomass was pyrolyzed at 300 and 700 °C for 2 h under oxygen-limited conditions in a muffle furnace. The pyrolysis heating rate was 10 °C/min. Then, the obtained biochar sample was passed through a 0.355 mm sieve again.

HCl, NaOH and HNO_3_ (2 M) were used separately to modify the original biochar, which were common acidic, alkaline, and oxidative modification process, respectively^45,46^. The modifier and original biochar were added at a solid-to-liquid ratio of 1:20 (weight/volume). The mixed samples were placed into a glass beaker and stirred on a magnetic stirrer for 4 h at 500 rpm, after which they were left undisturbed for 24 h. After repeating this modification process to ensure complete modification of the biochar, the obtained samples were washed completely with deionized water, dried at 80 °C for 24 h, and finally passed through a 0.355 mm sieve.

### Synthesis of biochar-supported ZVI

Ball milling was used to prepare the synthesis of the modified biochar-supported ZVI materials, which was environment-friendly, simple operation and easy mass production method^13,47^. A total of 12.00 g of biochar and a certain amount of ZVI (0.12, 0.24, 0.40, 1.20, 2.4, and 6.0 g) were placed into an agate ball mill jar with different mass ratios (ZVI:biochar = 1:100, 1:50, 1:30, 1:10, 1:5, and 1:2, respectively). Then, approximately 314 g of zirconia ball grinding beads were added (the mass ratio of 2 mm to 1 mm zirconia balls was approximately 1:8). The material was milled at 300 rpm for 6 h. The obtained composite materials were labeled XX-T-Y, with XX denoting the modifier (CK, NaOH, HNO_3_, or HCl), T denoting the pyrolysis temperature (300 or 700), and Y denoting the ZVI:biochar mass ratio (Fe100, Fe50, Fe30, Fe10, Fe5, or Fe2). For example, NaOH-700-Fe10 indicates ZVI supported on NaOH-modified biochar pyrolyzed at 700 °C with a 1:10 mass ratio (ZVI:biochar). All synthesized composite materials were stored in 50 mL centrifuge tubes.

### Experimental procedures

#### Removal kinetics experiments

The kinetics experiments were conducted under anaerobic conditions. Before the experiments, all water was purged with N_2_ for 1 h to completely remove oxygen. Twenty-two-milliliter glass vials with Teflon-lined screw caps containing 20 mL of deoxygenated water and 0.020 g of test material were used to conduct the kinetics experiments. Na_2_SO_4_ (0.01 M) was added as the background electrolyte to simulate groundwater. Nitrobenzene was added to each glass vial to reach a concentration of 100 mg/L. The glass vials were placed in an oscillator and shaken at 200 rpm and 25 ± 1 °C. A blank control experiment was conducted without any additional materials. The samples were analyzed after 5 min, 10 min, 15 min, 30 min, 60 min, 3 h, 6 h, 12 h, 24 h, 48 h and 72 h of shaking. The supernatants of all samples to be tested were filtered through a 0.22 µm filter.

#### Nitrobenzene desorption from contaminated soil

A total of 1.0 g of aged nitrobenzene-contaminated soil (332.3 ± 5.6 mg/kg) and different amounts of biochar materials (soil weight:material weight: 100:1, 100:3) were added to 22 mL glass vials equipped with Teflon-lined screw caps containing 20 mL of deoxygenated water. Na_2_SO_4_ (0.01 M) was added to the deoxygenated water as the background electrolyte to simulate groundwater. Then, the glass vials were placed in an oscillator and shaken at 200 rpm and 25 ± 1 °C for 24 h. The supernatant was subsequently filtered through a 0.22 µm filter for further analysis.

#### Nitrobenzene removal experiment in anaerobic soil

A total of 100.0 g of aged nitrobenzene-contaminated soil (2.9 ± 0.07 mg/kg) and different amounts of biochar materials (soil weight:material weight: 100:1, 100:3) were added to 250 mL brown glass sample bottles and mixed homogeneously. Deoxygenated water was added to the glass bottles until the water surface was flush with the bottle mouth, after which the bottle cap was tightened to ensure that the bottle was securely closed. Simultaneously, ℽ radiation sterilization (50 kGy) treatment was carried out for the blank control. All the sample bottles were placed in a 25 °C incubator for 30 days in the dark. After culture, soil and water samples were collected from the bottles to determine the concentration of nitrobenzene. Other soil samples were taken for soil microbial analysis. The treatments were labeled XX-T-Y-1% and XX-T-Y-3%, with 1% and 3% denoting the mass addition amount of composite material.

#### Determination of nitrobenzene concentration

Soil samples (2.0 g) were added to 8 mL glass vials equipped with Teflon-lined screw caps, and 4 mL of methanol was added for extraction. The vials were vortexed for 1 min, subjected to ultrasonic extraction for 30 min, held for 5 min, and centrifuged at 3000 rpm for 5 min. The supernatant was filtered through a 0.22 µm filter and transferred to a 2 mL chromatography bottle for further analysis.

The concentration of nitrobenzene in the aqueous phase was determined using an ultra-performance liquid chromatography (UPLC) system (Waters, USA, H-grade) with a BEH-C18 chromatographic column (2.1 × 50 mm, 1.7 μm), and the wavelength of the UV detector was 254 nm. The mobile phase consisted of methanol and water at a ratio of 60:40 and a flow rate of 0.2 mL/min.

### Characterization

The morphology and elemental distribution of the biochar samples were characterized using scanning electron microscopy (SEM, Hitachi, Japan, SU8600) and energy dispersive X-ray spectroscopy (EDS). The changes in functional groups in the biochar samples were analyzed by Fourier transform infrared (FTIR) spectroscopy (Bruker, Germany, INVENIO S). The crystal structure of the biochar samples was analyzed by X-ray diffraction (XRD; Rigaku, Japan; SmartLab-9). The pore structure of the biochar samples was analyzed by a Brunauer–Emmett–Teller (BET) analyzer (MicrotracBEL, Japan, BELSIRO-MAX). Thermo analysis (TGA) were conducted using a thermogravimetric analyzer (Netzsch, Germany, STA 449 F5) under flow of nitrogen with a heating rate of 10 °C min^−1^, at the range of 30–1100 °C.

### Soil microbial community analysis

To investigate the effects of the modified biochar-supported ZVI materials on the anaerobic soil microbial community associated with nitrobenzene removal and detoxification, the microbial community in selected treatment soils was analyzed using previous methods^48^. Bacterial DNA in soil was extracted using a Fast DNA Spin Kit following the manufacturer's instructions, and three replicates were used in each sample for quantification and qualification. The DNA quality and concentration were analyzed with a Nano-Drop 2000. In this study, primers 338F (5'-ACTCCTACGGGAGGCAGCAG-3') and 806R (5'-GGACTACHVGGGTWTCTAAT-3') were used for PCR amplification. Sequencing libraries were paired-end sequenced (2 × 300) on the Illumina MiSeq platform according to standard protocols. The datasets generated and analyzed in this study are available from the NCBI SRA repository (BioProject accession number: PRJNA1112572). The richness and diversity of the microbial community were used to analyze the α diversity of the bacterial community in these treatments. The detailed calculation steps for the microbial community analysis were completed by Majorbio Bio-Pharm Technology Co., Ltd. (Shanghai, China).

### Data analysis

All the experimental data were obtained from triplicate parallel samples to ensure accuracy. Both the blank samples and the recovered samples met the quality standards, which ensured the reliability of the method. The differences among the treatments were tested by independent t tests using SPSS 20.0 software. The significance for all statistical analyses was set to *p* = 0.05.

## Results and discussion

### Characterization of biochar composite materials

**Figure 1:**
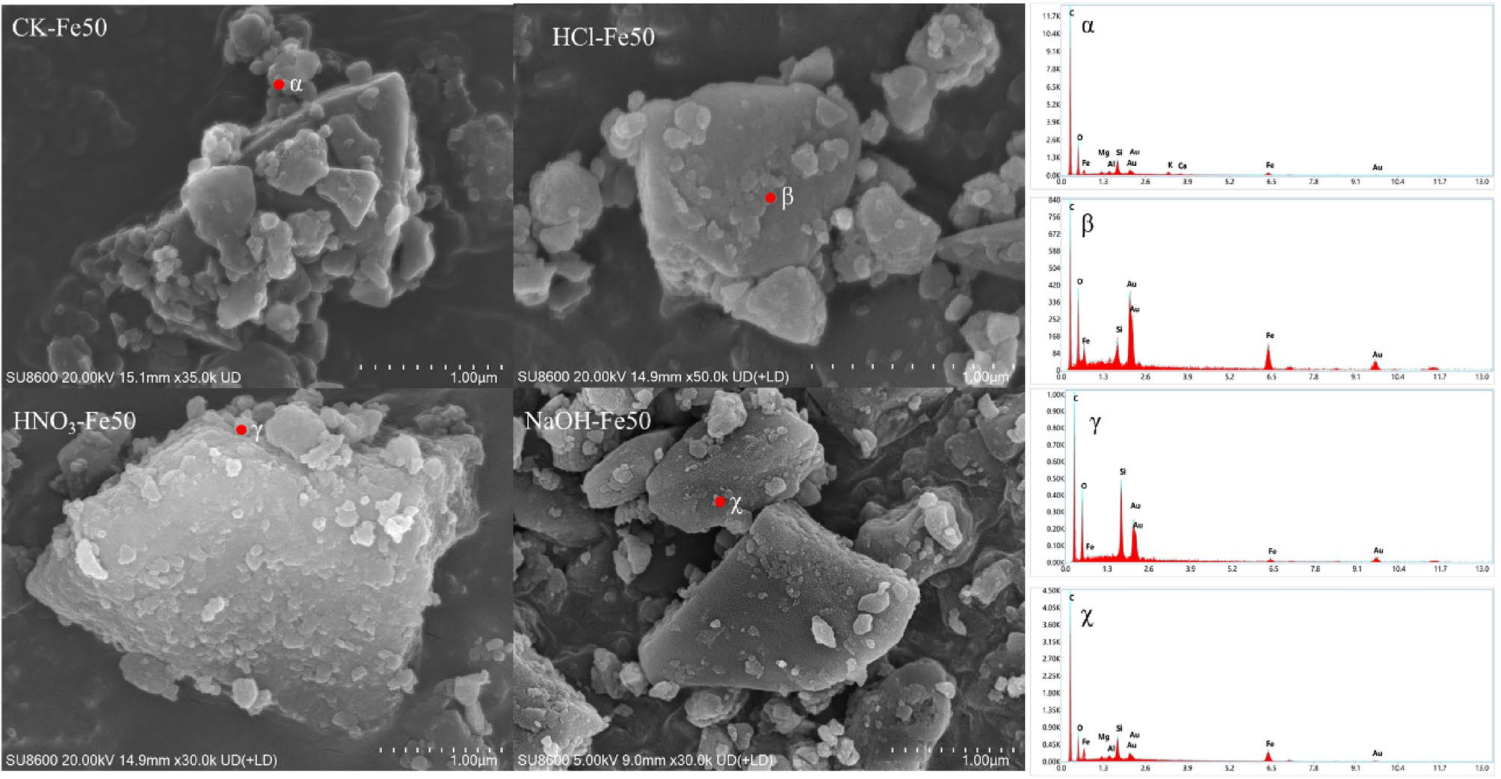
SEM–EDS analysis of different biochar composite materials.

SEM‒EDS images of the biochar and biochar composite materials are shown in Fig. 1 and Fig. S1. The biochar composite materials exhibited a sheet-like structure with a rough surface and pore structure, but the pore structure of the 300 °C biochar did not form well, indicating that the 700 °C biochar had a greater specific surface area and loading capacity for ZVI. After ball milling, the particle size of the biochar composite materials decreased remarkably compared with that of the original biochar, which could provide excellent attachment sites for ZVI particles. According to the SEM results, the particle size of the biochar composite materials was approximately 1 μm, and ZVI was uniformly attached to the biochar surface, which further suggested that the biochar composite materials were prepared successfully. This dispersion behavior could greatly improve the reactivity of ZVI and reduce aggregation. This result was similar to that obtained for nZVI/BC synthesized by liquid-phase reduction^49^.

The BET results for the different biochar composite materials are presented in Table S1. The highest total, micropore and mesopore surface areas of NaOH-700-Fe50 were 394.36, 505.81 and 162.38 m^2^/g, respectively, followed by NaOH-700-Fe30 and HCl-700-Fe50, while, those of CK-700-Fe10 were 221.6, 226.2 and 185.7 m^2^/g, respectively. Ball milling increased the specific surface area of the biochar composite materials, which was attributed to the squeezing action of the grinding balls^50^. The loading of ZVI decreased the specific surface area of biochar composite materials, which might be due to the occupation of samples micropores by ZVI. The attachment of ZVI changed the morphology of the samples, leading to the formation of larger pores and a fluffy structure^51^.

**Figure 2:**
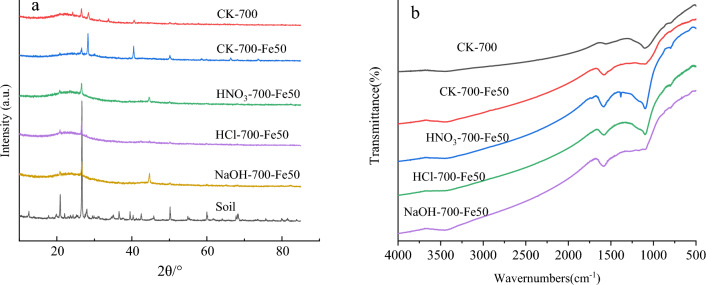
XRD patterns and FTIR spectra of biochar and different biochar composite materials.

The XRD patterns of the biochar and biochar composite materials are shown in Figs. 2a and S2. 2θ = 26.5° was the characteristic diffraction peak of biochar, and 2θ = 44.7°, 65.0°, and 82.3° were the characteristic diffraction peaks of the crystal planes of Fe. Diffraction peaks corresponding to biochar and Fe(0) were detected, indicating the successful synthesis of biochar composite materials. Meanwhile, the diffraction peak intensity of Fe gradually increased with the addition of ZVI (Fig. S2).

The FTIR spectra of the biochar and biochar composite materials are shown in Figs. 2b and S3. -OH and N–H groups^52^, C = O and C = C groups^53^, and C = C and C-O groups^54^ were the conventional functional groups of the biochar composite materials and were present at 3441 cm^−1^, 3129 cm^−1^, 1620 cm^−1^, 1400 cm^−1^ and 1105 cm^−1^. The peaks at 480 cm^−1^ and 1580 cm^-1^ for the biochar composite materials were attributed to Fe–O tensile vibrations, which further indicated the successful synthesis of biochar and ZVI^55,56^.

Fig. S6 showed the thermal degradation behavior of wheat straw and biochar. There is a gradual decrease in weight loss from 30 °C to 1100 °C, indicating the decomposition of wheat straw with the temperature increase. The dehydration of wheat straw (Fig. S6a) took place between 30 °C to 135 °C^57^, and devolatilization got started at 250–400 °C which strongly implied dehydration reaction and confirmed the decomposition of hemicelluloses and cellulose^58^. Then slow weight loss began at 400–700 °C, indicating a thermally stable char formation develops, which was due to similar thermal degradations of hemicellulose, cellulose, and lignin^59^. For 300 °C biochar materials (Fig. S6b and d), slight weight loss began at 30–300 °C, and rapid weight loss was observed at 300–500 °C, which might be the decomposition of hemicelluloses and cellulose. For 700 °C biochar materials (Fig. S6c, e, f, g, and h), the slow weight loss was observed at 30–1100 °C, indicating that 700 °C biochar materials had higher thermal stability, than 300 °C biochar materials and wheat straw.

### Kinetics and desorption of nitrobenzene on biochar composite materials

To investigate whether these biochar composite materials could remove nitrobenzene via a reduction process, the removal of nitrobenzene by modified biochar-supported ZVI composite materials with ZVI/C ratios of 1/10 and 1/2 was conducted as shown in Fig. S4. The low-iron-loading biochar treatments (ZVI/C ratio = 1/10) resulted in greater nitrobenzene removal, which was probably due to the strong adsorption of nitrobenzene by the biochar, resulting in higher enrichment of nitrobenzene. Under the same ZVI loading, HNO_3_ modification inhibited the removal of nitrobenzene; however, HCl and NaOH modification enhanced the removal of nitrobenzene. This might be because the HNO_3_-modified biochar matrix inhibited the sorption of nitrobenzene, while HCl and NaOH modification enhanced nitrobenzene sorption^60^. When the iron loading ratio was 1/10, no intermediates such as aniline were detected after the experiment, possibly due to the instability or trace amount of aniline. However, when the iron loading amount was 1/2, aniline was detected, and the HCl-modified biochar composite material (HCl-700-Fe2) had the highest concentration of aniline (1.33 mg/L), followed by NaOH-700-Fe2 (0.52 mg/L), CK-700-Fe2 (0.08 mg/L), and HNO_3_-700-Fe2 (< 0.01 mg/L). The reduction efficiencies of nitrobenzene in HCl-700-Fe2 and NaOH-700-Fe2 were 10.4% and 3.9%, respectively. These results indicated that the removal of nitrobenzene by these biochar-ZVI materials mainly relied on adsorption and that HCl and NaOH modification promoted the reduction of nitrobenzene, while HNO_3_ modification hindered the reduction of nitrobenzene. Moreover, it could be inferred that at an Fe/C ratio of 1/50, no aniline could be detected during the removal process.

Desorption experiments were performed to investigate whether the addition of biochar composite materials could affect the desorption of nitrobenzene from contaminated soil. The concentration of nitrobenzene in aqueous solution is shown in Fig. S5. After desorption equilibrium was reached in the systems with high concentrations of nitrobenzene-contaminated soil amended with different biochar materials, the highest concentration of nitrobenzene was observed in the control at 18.9 mg/L, followed by that in the BC-300–1% (14.5 mg/L) and BC-300-Fe50-1% (11.3 mg/L) treatments, which included biochar prepared at 300 °C. Moreover, the concentrations of nitrobenzene in the 700 °C biochar composite treatments were significantly lower than those in the soil and 300 °C biochar composite treatments. The desorption amount of nitrobenzene decreased as the amount of added amendments increased. Compared to that in the CK-700-Fe50-3% treatment (0.02 mg/L), the desorption amount of nitrobenzene decreased in the NaOH-700-Fe50-3% and HCl-700-Fe50-3% treatments (< 0.01 mg/L) but increased in the HNO_3_-700-Fe50-3% treatment (0.19 mg/L), demonstrating that HCl and NaOH modification inhibited the desorption of nitrobenzene and that HNO_3_ promoted the desorption of nitrobenzene.

### Removal and residual content of nitrobenzene

**Figure 3:**
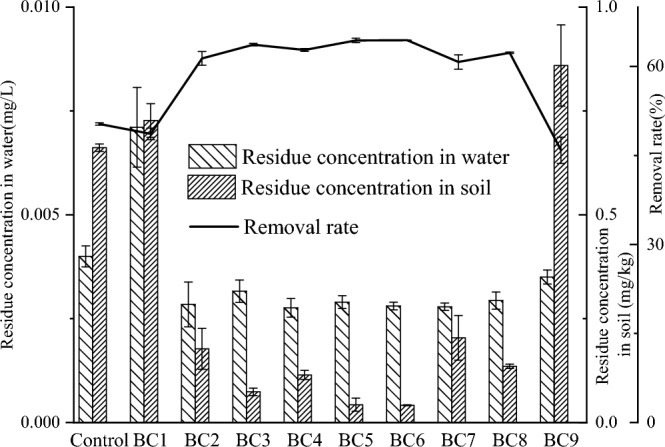
Removal rates of nitrobenzene in different treatments. Control: soil; BC1: CK-300-Fe50-1%; BC2: CK-700-Fe50-1%; BC3: CK-700-Fe50-3%; BC4: HNO_3_-700-Fe50-3%; BC5: HCl-700-Fe50-3%; BC6: NaOH-700-Fe50-3%; BC7: CK-700–1%; BC8: CK-700–3%; BC9: CK-300–1%.

The results for the removal of nitrobenzene in soil and the residual concentrations of nitrobenzene in water and soil are presented in Fig. 3. The removal of nitrobenzene from the contaminated soil followed the order BC6 > BC5 > BC3 > BC4 > BC8 > BC2 > BC7 > Control > BC1 > BC9. The highest removal of nitrobenzene in soil was observed in the BC6 treatment (64.42%), followed by that in the BC5 treatment (64.39%), which was markedly higher than that in the control (50.30%). These results indicated that HCl and NaOH modification enhanced the removal of nitrobenzene from the biochar composite materials. The removal of nitrobenzene in the BC1 and BC9 treatments was lower than that in the control, indicating that the 700 °C-modified biochar composite material could promote the removal of nitrobenzene in soil; however, the 300 °C-modified biochar composite material inhibited the removal of nitrobenzene in soil. In previous studies, nitrosobenzene and phenylhydroxylamine were commonly considered reduction intermediates of nitrobenzene^3,36^. However, these intermediates were not detected in this study, which might have occurred due to the instability of these intermediates or because they were present in trace amounts. Compared with those of BC2 and BC3, a greater amount of composite material was added in BC7 and BC8, resulting in an increase in the removal rate of nitrobenzene.

As shown in Fig. 3, the residual concentrations of nitrobenzene in water in the BC1 and BC9 treatments were greater than those in the control, indicating that the 300 °C-modified biochar composite materials promoted the desorption of nitrobenzene from soil. The residual concentrations of nitrobenzene in water in the other treatments were lower than those in the control, indicating that the 700 °C-modified biochar composite materials inhibited the desorption of nitrobenzene from soil or promoted the removal of nitrobenzene in the aqueous phase. The highest residual nitrobenzene concentration in soil was observed in the BC9 treatment, followed by the BC1 and control treatments. The residual nitrobenzene concentration in the soil in the other treatments was significantly lower than that in the control. These results demonstrated that the 700 °C-modified biochar composite materials promoted the removal of nitrobenzene in soil, while the 300 °C-modified biochar composite materials inhibited nitrobenzene removal. Moreover, the residual content of nitrobenzene in the BC6 treatment was the lowest, followed by that in the BC5 treatment, demonstrating that HCl and NaOH modification enhanced the soil nitrobenzene removal effect of the biochar composite materials. Moreover, the residual ratios of nitrobenzene in the soil and water phases were analyzed, as shown in Fig. S7. The residual ratio of nitrobenzene in the soil phase exceeded 80%, demonstrating that the removal of nitrobenzene mainly occurred in the soil rather than in the aqueous phase. The lowest residual ratio of nitrobenzene in the soil was observed in the BC5 treatment, followed by that in the BC6 treatment, probably due to the enhanced removal of nitrobenzene in the soil.

### Microbial community

The soil microbial communities in the selected treatment groups were analyzed to investigate the effects of the microbes on nitrobenzene removal. The microbial diversity indices are shown in Table S2. The coverage index of the treatments was nearly 1, indicating that the number of bacterial sequences fully represented the number of bacterial communities (Table S2). Compared with those in the control, the Shannon, Shannoneven and Chao 1 indices increased in the other treatments, indicating that the biochar composite materials could increase the diversity and community evenness of the bacterial community in the soil. High biodiversity and community evenness can enhance ecological stability, thereby endowing a system with high resistance to environmental stress and toxicity^61^. Compared with biochar alone, the biochar composite materials had no significant effect on microbial diversity but resulted in better remediation efficiency. Thus, further analysis of the structure and composition of the bacterial communities is needed to investigate the effect of microorganisms on organic pollutant remediation.

Principal coordinate analysis (PCoA) of the microbial communities was performed, as shown in Fig. S8. The contribution of PCoA to the microbial community composition was 82.59%, which could explain the dynamics of the microbial community. In the PCoA matrix, the position of the control was different from that of the other treatments, indicating that the addition of biochar composite materials could change the microbial community composition. Among the different treatments, significant differences were observed in the relative abundance of microorganisms at the phylum and family levels, as shown in Figs. S9 and S10. The main bacterial phyla were Proteobacteria, Firmicutes, Actinobacteriota, Gemmatimonadota, Bacteroidota, Chloroflexi, Acidobacteriota, Myxococcota, Patescibacteria, and Cyanobacteria. At the phylum level, Firmicutes, Proteobacteria, Gemmatimonadota, and Actinobacteriota were the main taxa of the control, with relative abundances of 51.3%, 21.5%, 8.8%, and 5.5%, respectively. Firmicutes was the dominant phylum in the control, with a relative abundance of 51.3%, which was greater than that in the other treatments. Proteobacteria was the dominant phylum in the addition treatments, and the highest abundance was observed in BC8 at 44.5%, while the control had the lowest abundance at 21.5%. Similarly, in a previous study, Fe_3_O_4_ and nZVI treatment resulted in Proteobacteria being the dominant phylum^62^. The biochar composite materials increased the relative abundance of Proteobacteria but significantly decreased the relative abundance of Firmicutes. All the treatments increased the relative abundance of Actinobacteriota, and the highest increase in abundance was obtained in the BC6 treatment, reaching 10.7%. In some studies, in sediments and anaerobic soils, Firmicutes and Proteobacteria were the dominant Fe(III)-reducing bacteria^62,63^. In addition, there are several sulfate-reducing bacteria in the phylum Proteobacteria, such as Desulforhabdus, Desulfovibrio, and Desulfuromonas^49,64^. These results demonstrated that the biochar composite materials could change the relative abundance of microorganisms and stimulate the enrichment of Fe-reducing and sulfate-reducing bacteria.

At the class level (Fig. S10), the dominant taxa in the control were *Bacilli*, *Gammaproteobacteria*, *Symbiobacteriia*, and *Gemmatimonadetes*, accounting for 30.7%, 18.4%, 14.5%, and 7.6%, respectively, which was consistent with the results at the phylum level (Fig. S9). The relative abundances of *Gammaproteobacteria* and *Alphaproteobacteria* were markedly enhanced in comparison to those in the control, indicating that *Alphaproteobacteria* and *Gammaproteobacteria* could promote the removal of nitrobenzene after the addition of biochar composite materials. Moreover, the relative abundance of *Actinobacteria*, which is involved in nitrobenzene biodegradation, was significantly enhanced to 9.0% in BC6 treatment compared to the control (4.2%)^65^. Furthermore, *Bacteroidia* was stimulated in the BC3, BC5, and BC6 treatments (5.1–5.9%) compared to the control (3.6%), while inhibition was observed in the BC4 treatment (2.2%). Bacteria in the *Bacteroidia* family can be highly resilient to pollution and sterilization disturbances^66^.

**Figure 4:**
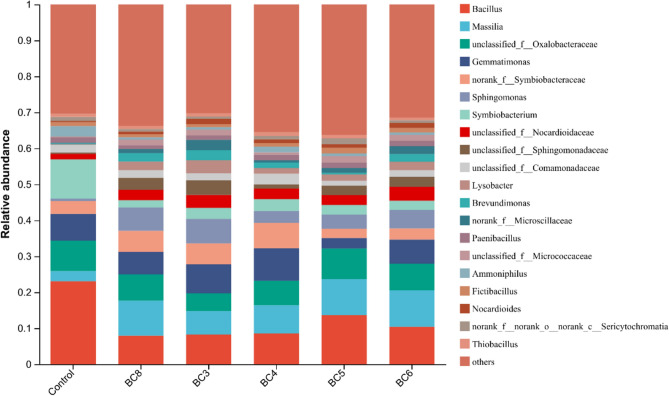
Bacterial community structure of the soil at the genus level. Control: soil; BC8: CK-700–3%; BC3: CK-700-Fe50-3%; BC4: HNO_3_-700-Fe50-3%; BC5: HCl-700-Fe50-3%; BC6: NaOH-700-Fe50-3%.

At the genus level (Fig. 4), *Bacillus*, *Symbiobacterium*, *Oxalobacteraceae*, *Gemmatimonas*, and *Symbiobacteraceae* were the dominant taxa in the control. After the addition of the biochar composite materials, there was a significant change in the dominant genera compared to those in the control. For example, *Massilia* and *Sphingomonas* became the major genera after the addition of 3% biochar only. The relative abundances of the top five genera changed after the addition of the biochar composite materials. In the BC5 and BC6 treatments, the five dominant genera were *Bacillus*, *Massilia*, *Oxalobacteraceae*, *Gemmatimonas* and *Sphingomonas*. In the BC4 treatment, the five dominant genera were *Bacillus*, *Massilia*, *Oxalobacteraceae*, *Gemmatimonas* and *Symbiobacteraceae*. In the BC3 treatment, the five dominant genera were *Bacillus*, *Massilia*, *Gemmatimonas*, *Symbiobacteraceae* and *Sphingomonas*. The dominant genera in the biochar treatments differed from those in the biochar composite material treatments, which demonstrated that ZVI significantly changed the microbial community composition at the genus level^49^. *Massilia* are petroleum- and polycyclic aromatic hydrocarbon (PAH)-degrading bacteria^67,68^ that might be involved in the nitrobenzene degradation process. Moreover, biochar and the composite materials enhanced the relative abundance of *Sphingomonas*, which can degrade nitrobenzene^69,70^. Additionally, the abundance of *Bacillus*, which can degrade nitrobenzene, decreased after amendment addition^65,71,72^, possibly because *Bacillus* was not the main degrading bacteria in this study. *Nocardioidaceae* benefitted from nitrobenzene in the biochar- and composite material-amended soils, possibly because *Nocardioidaceae* are more ecologically competitive because of their high affinity for inorganic nitrogen and carbon substrates^73^. Due to the enrichment of nitrobenzene by the biochar materials with abundant pores and a large specific surface area, microorganisms with a reducing effect on nitroaromatic hydrocarbons were enriched, and the toxicity of nitrobenzene to microorganisms decreased^74^. When biochar was added, the relative abundance of *Sphingomonadaceae* increased; these bacteria are electroactive bacteria that can change the redox potential and biodegrade bisphenol A^75,76^. In a previous study, *Comamonadaceae* was shown to degrade aromatic compounds in a nitrate-reducing microbial community^77^. When biochar was added, the relative abundance of *Lysobacter* increased; this genus could degrade oil isolated from selected marine bacteria^78^. *Norank_f__Microscillaceae* was reported to have the ability of nitrification^79^.

### Removal mechanisms

Nitrobenzene can be adsorbed to the soil phase from the water phase, adsorbed by biochar composite materials, reduced by ZVI, and biodegraded by microorganisms in contaminated soil and water^80^. These processes could affect the environmental fate of nitrobenzene. According to the desorption results (Fig. S5), nitrobenzene could be transferred from the soil phase to the aqueous phase. Among the different biochar material treatments, the aqueous concentration of nitrobenzene in the treatments with 300 °C-pyrolyzed biochar composite materials was significantly higher than that in the treatments with 700 °C-pyrolyzed biochar composite materials, indicating that the materials prepared at 300 °C were not suitable for the removal of nitrobenzene in groundwater soil. Moreover, the HCl-700-Fe50 treatment had the lowest aqueous concentration of nitrobenzene, followed by the NaOH-700-Fe50 treatment. Therefore, the 700 °C-modified biochar composite materials benefited the enrichment of nitrobenzene from groundwater; this effect could further reduce the migration of nitrobenzene from the aqueous phase.

ZVI can remove nitrobenzene via a reduction process^81^. According to the nitrobenzene removal kinetics results, no intermediates, such as aniline, were detected with the addition of biochar composite materials when the Fe/biochar ratio was 1/10, which was probably due to the generation of trace amounts of aniline. When the Fe/biochar ratio was 1/2, a small amount of aniline was detected after the biochar composite materials were added, which indicated that these composite materials could reduce nitrobenzene; however, the reduction ability was related to the loading amount of ZVI. Thus, we inferred that no reduction occurred when the Fe/biochar ratio was 1/50. This excluded a reduction effect of the biochar composite materials in this simulated aquifer experiment and demonstrated that the removal of nitrobenzene was accomplished by degradation by the microbial community.

The microbial community is an important driver of soil organic pollutant removal, electron transfer and nutrient cycling^74,82,83^. An increase in the capacity of microorganisms for nitrobenzene degradation is the key to enhancing nitrobenzene removal. When biochar was supplemented with ZVI, the relative abundance of *Nocardioides*, which is involved in nitrobenzene degradation, increased^84^. NaOH and HCl modification enhanced the relative abundance of *Massilia*. HNO_3_ modification increased the relative abundances of *Gemmatimonas* and *Symbiobacteraceae* and decreased the relative abundance of *unclassified_f__Sphingomonadaceae*. The functional microorganisms involved in nitrobenzene degradation varied depending on the treatment, and these changes might be due to changes in the soil environment. First, the biochar treatment increased the relative abundances of *Massilia*, *Sphingomonas*, and *Symbiobacteraceae*. *Massilia* might indirectly participate in the degradation of nitrobenzene by participating in the degradation of the benzene ring^67,68^. The enrichment of *Sphingomonas*, which can degrade nitrobenzene, could be ascribed to the addition of biochar. The abundance of *Symbiobacteraceae*, which has been reported to be involved in the nitrogen cycle, increased after the addition of biochar^85^. Second, ZVI increased the relative abundances of *unclassified_f__Nocardioidaceae* and *unclassified_f__Sphingomonadaceae*, indicating that the ecological competitiveness of the carbon and nitrogen cycles increased and that the redox potential changed^73,75,76^. Modification also changed the relative abundance of related degrading bacteria, although there was no obvious pattern. According to previous reports, *Bacillus* and *Symbiobacterium* were the main microbial communities and exhibited significantly higher relative abundances in the control treatment. *Bacillus* species are typical degraders of nitrobenzene^71^, and *Symbiobacterium* species participate in the nitrogen cycle^86^. These results demonstrated that biochar might promote the removal of nitrobenzene by changing the types of nitrobenzene degraders and microbial communities involved in nutrient cycling. Moreover, the relative abundances of degrading bacteria became more balanced after the addition of HCl- and NaOH-modified biochar composite materials, which might be the reason for the increased removal of nitrobenzene.

**Figure 5:**
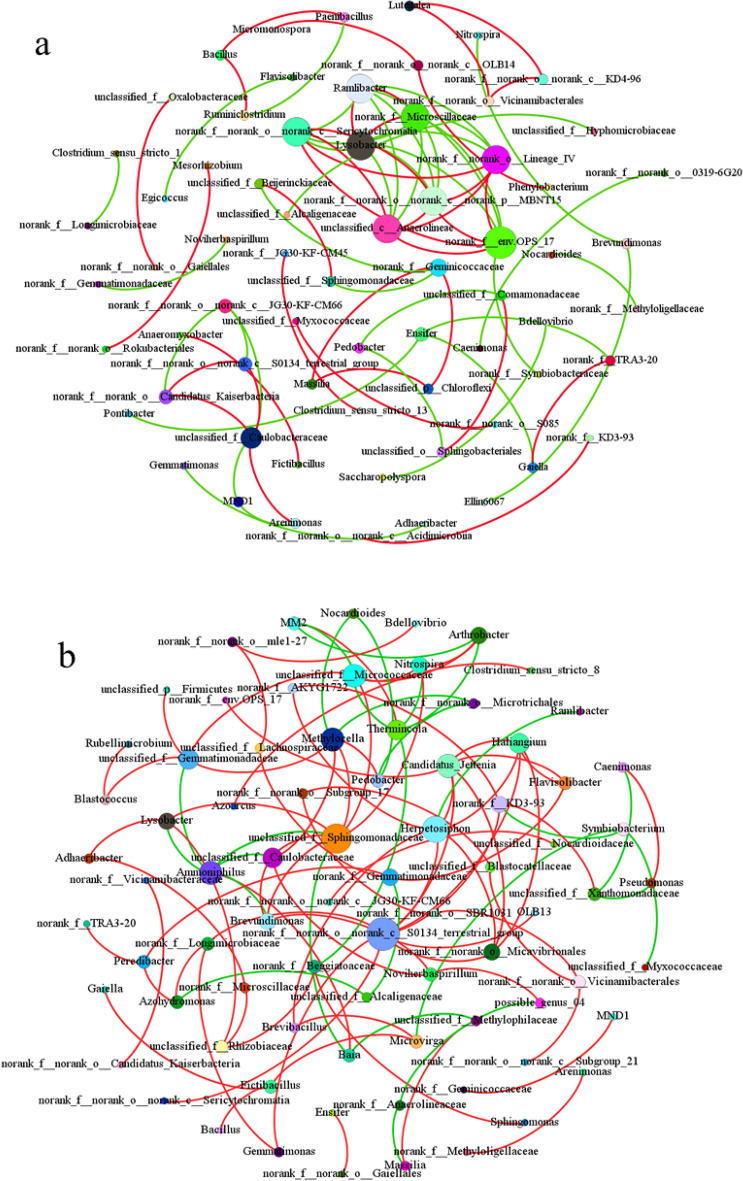
Co-occurrence network of soil microbiota in non-ZVI-treated samples (**a**) and ZVI-treated samples (**b**) at the genus level.

The co-occurrence network of soil bacteria at the phylum level with the addition of ZVI is shown in Fig. S11. In the no-ZVI treatment, the phyla Cyanobacteria, Elusimicrobiota and MBNT15 were the core nodes microbiota, and a positive correlation was observed. After ZVI addition, the co-occurrence network of soil microbiota was enriched, leading to increased stability of the microbial community structure. Gemmatimonadota and Planctomycetota were the core microbiota and exhibited a positive correlation, while Firmicutes, Actinobacteriota and Bacteroidota exhibited negative correlations. These results indicated that ZVI enriched the microbial community structure and increased the competition between soil microorganisms at the phylum level. When ZVI was introduced, the microbial community structure in the soil would be changed, including promotion of the proliferation of microorganisms which might have degradable capacity, or inhibition of other microorganisms which have sensitively to ZVI^87^. This resulted in a significant proliferation of adaptable microorganisms and the inhibition of other microorganisms, potentially leading to changes in microbial community structure at the phylum level. Similar research has been shown that ZVI increased Chloroflexi, Firmicutes and Euryarchaeota by 2.46, 1 and 3.53 times, respectively, whereas decreased Bacteroidetes, Synergistetes, Proteobacteria and Caldiserica^88^. At the genus level (Fig. 5), *unclassified_c__Anaerolineae*, *norank_f__Microscillaceae*, and *norank_f__envOPS_17* were the core microbial genera, and the majority were negatively correlated (52%). However, after ZVI addition, the core microbial genera changed to *norank_f__norank_o__norank_c__S0134_terrestrial_group*, *unclassified_f__Sphingomonadaceae*, and *Herpetosiphon,* which could maintain the stability of the microbial community and had a positive correlation (73.8%). These results indicated that symbiotic interactions within microbial genera increased after ZVI addition, while competition between soil microbial genera decreased.

**Figure 6:**
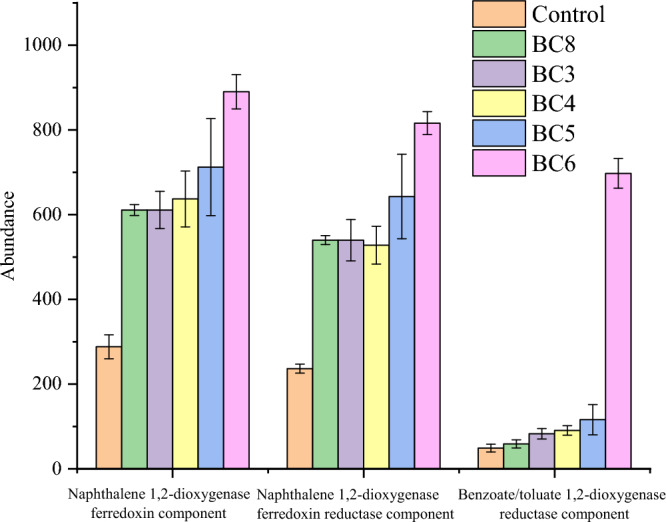
Potential related degradation genes in the treatments. Control: soil; BC8: CK-700–3%; BC3: CK-700-Fe50-3%; BC4: HNO_3_-700-Fe50-3%; BC5: HCl-700-Fe50-3%; BC6: NaOH-700-Fe50-3%.

Functional gene analysis of microbial communities can provide useful information on nitrobenzene biodegradation by those communities. Here, PICRUSt was used to analyze the interactions between nitrobenzene removal and microbial communities as a powerful tool for predicting functional composition. The degradation of nitrobenzene can start with initial oxidization by dioxygenase or with reduction by nitrobenzene nitroreductase. These processes could be described as direct metabolic pathways of nitrobenzene degradation. With the addition of biochar composite materials, the functional genes associated with dioxygenase, such as the naphthalene 1,2-dioxygenase ferredoxin component, naphthalene 1,2-dioxygenase ferredoxin reductase component, and benzoate/toluate 1,2-dioxygenase reductase component, were upregulated (Fig. 6). However, under anaerobic soil conditions, aerobic degradation driven by related functional genes might not be the main removal pathway of nitrobenzene. The reductive degradation of nitrobenzene might be the key removal pathway in anaerobic groundwater environments. However, compared with those in the control, the expression levels of the nitrobenzene nitroreductase gene in the biochar composite material treatments were not significantly different (Fig. S12), and the expression levels of the hydroxylamine reductase and nitrite reductase (NO-forming)/hydroxylamine reductase genes were upregulated. Moreover, among the modified biochar composites, HNO_3_ modification (BC4) decreased the expression of hydroxylamine reductase genes. Nitrobenzene plays important roles in nitrate–N transformation; for example, nitrobenzene preserves ammonia nitrogen and changes the nitrogen cycle during the aerobic–anoxic transition process^89^. The functional genes of nitrate reductase were upregulated, which probably affected the degradation of nitrobenzene, and the upregulation of the dihydroorotate dehydrogenase electron transfer subunit and nitrate reductase electron transfer subunit (Fig. S12) demonstrated that the electron transfer ability of the nitrobenzene degradation process improved. These results demonstrated that the biochar composites could also promote the regulation of nitrogen and electron transfer processes to increase nitrobenzene degradation. There was no obvious pattern of changes in the expression of these genes during modification; however, NaOH generally upregulated these functional genes. Therefore, the mechanism of nitrobenzene removal in anaerobic soil environments likely involves comprehensive effects on nitrobenzene- degrading microbes, reduction-degrading functional genes and electron transfer via biochar composite materials. NaOH modification had the greatest effect on these processes.

## Conclusion

The aim of this study was to evaluate the effectiveness of biochar composite materials for the removal of nitrobenzene in anaerobic soil. These results showed that nitrobenzene removal by NaOH-700-Fe50 was significantly enhanced compared to the control. The introduction of 700 °C biochar composite materials strongly inhibited the desorption of nitrobenzene from soil and increased the removal of nitrobenzene. Nitrobenzene removal was attributed mainly to degradation by microbial communities, upregulation of functional genes, and electron transfer. These findings suggested that NaOH-700-Fe50 is a promising composite material for in situ remediation of nitrobenzene-contaminated groundwater soil.

### Supplementary Information

Supplementary Information.

## Data Availability

Data will be made available on request. The datasets generated and analyzed in this study are available from the NCBI SRA repository (SRA accession number: PRJNA1112572) following publication, (https://www.ncbi.nlm.nih.gov/sra/PRJNA1112572).
