# Supplementary material for: Responses of nitrobenzene removal performance and microbial community by modified biochar supported zerovalent iron in anaerobic soil
Source: Sci Rep. 2024 Jul 24;14:17078. doi: 10.1038/s41598-024-67301-5 (PMC11269609; doi:10.1038/s41598-024-67301-5)
Supplement: Supplementary file 1 — Supplementary Information. [file 41598_2024_67301_MOESM1_ESM.docx]

**Supplementary material for removal of nitrobenzene by modified biochar-supported zerovalent iron in anaerobic soil: Performance and microbial community**

Lu Hainan^1^, Li Peng^1,2^, Li Qingqing^1^, Liu Fang^1^, Zhou Dong^1^, Huang Shenfa^3^, Yang Jie^1*^, Li Zhiheng^4^

^1^ *State Environment Protection Engineering Center for Urban Soil Contamination Control and Remediation, Shanghai Academy of Environmental Sciences, Shanghai 200233, China*

^2^ *School of Environmental Science and Engineering, Donghua University, Shanghai 201620, China*

^3^ *Shanghai Technology Center for Reduction of Pollution and Carbon Emissions, Shanghai 200235, China*

^4^ *School of Environmental Science and Engineering, Key Laboratory of Solid Waste Treatment and Recycling of Zhejiang Province, Zhejiang Gongshang University, Hangzhou 310018, Zhejiang Province, China*

^*^ Corresponding author

Tel: +86-021-64085119 Fax: +86-021-54973318

E-mail: [yangj@saes.sh.cn](mailto:yangj@saes.sh.cn)

| **Content** | |
| --- | --- |
| **15 pages (including title page and references)** | **Pages** |
| 3 Tables (Table S1-S5) | S2-S3 |
| 4 Figures (Figure S1-S11) | S4-S15 |

Table S1 Total, micropore, and mesoporous surface area of modified biochar composites

| BC | TSA  （m^2^/g） | MiSA  （m^2^/g） | MeSA  （m^2^/g） | APD  （nm） |
| --- | --- | --- | --- | --- |
| ZVI | 2.66 | 1.13 | 3.81 | 9.30 |
| CK-700 | 221.60 | 226.20 | 185.74 | 2.07 |
| CK-700-Fe10 | 163.28 | 186.12 | 74.32 | 2.87 |
| CK-700-Fe30 | 207.40 | 237.80 | 180.53 | 2.76 |
| CK-700-Fe50 | 289.66 | 328.02 | 161.58 | 2.28 |
| HNO_3_-700-Fe10 | 181.14 | 194.65 | 133.76 | 2.92 |
| HNO_3_-700-Fe30 | 267.54 | 315.61 | 206.03 | 2.51 |
| HNO_3_-700-Fe50 | 282.66 | 368.69 | 134.03 | 2.64 |
| HCl-700-Fe10 | 229.26 | 264.38 | 146.66 | 2.57 |
| HCl-700-Fe30 | 348.88 | 458.66 | 165.65 | 2.27 |
| HCl-700-Fe50 | 361.19 | 466.06 | 156.84 | 2.17 |
| NaOH-700-Fe10 | 252.83 | 300.35 | 105.30 | 2.45 |
| NaOH-700-Fe30 | 374.98 | 481.23 | 172.12 | 2.39 |
| NaOH-700-Fe50 | 394.36 | 505.81 | 162.38 | 2.25 |

TSA: total surface areas, MiSA: micropore surface areas, MeSA: mesopore surface areas, APD: average pore diameter

Table S2 Indexes of microbial diversity

| BC | Shannon | Shannoneven | Chao | Coverage |
| --- | --- | --- | --- | --- |
| Control | 3.504 | 0.652 | 216.49 | 1.000 |
| BC8 | 3.984 | 0.731 | 234.89 | 0.999 |
| BC3 | 3.926 | 0.725 | 226.21 | 1.000 |
| BC4 | 3.958 | 0.718 | 249.18 | 0.999 |
| BC5 | 4.074 | 0.731 | 266.80 | 0.999 |
| BC6 | 3.963 | 0.726 | 236.34 | 1.000 |

Control: Soil; BC8:CK-700-3%; BC3:CK-700-Fe50-3%; BC4:HNO_3_-700-Fe50-3%; BC5:HCl-700-Fe50-3%; BC6:NaOH-700-Fe50-3%


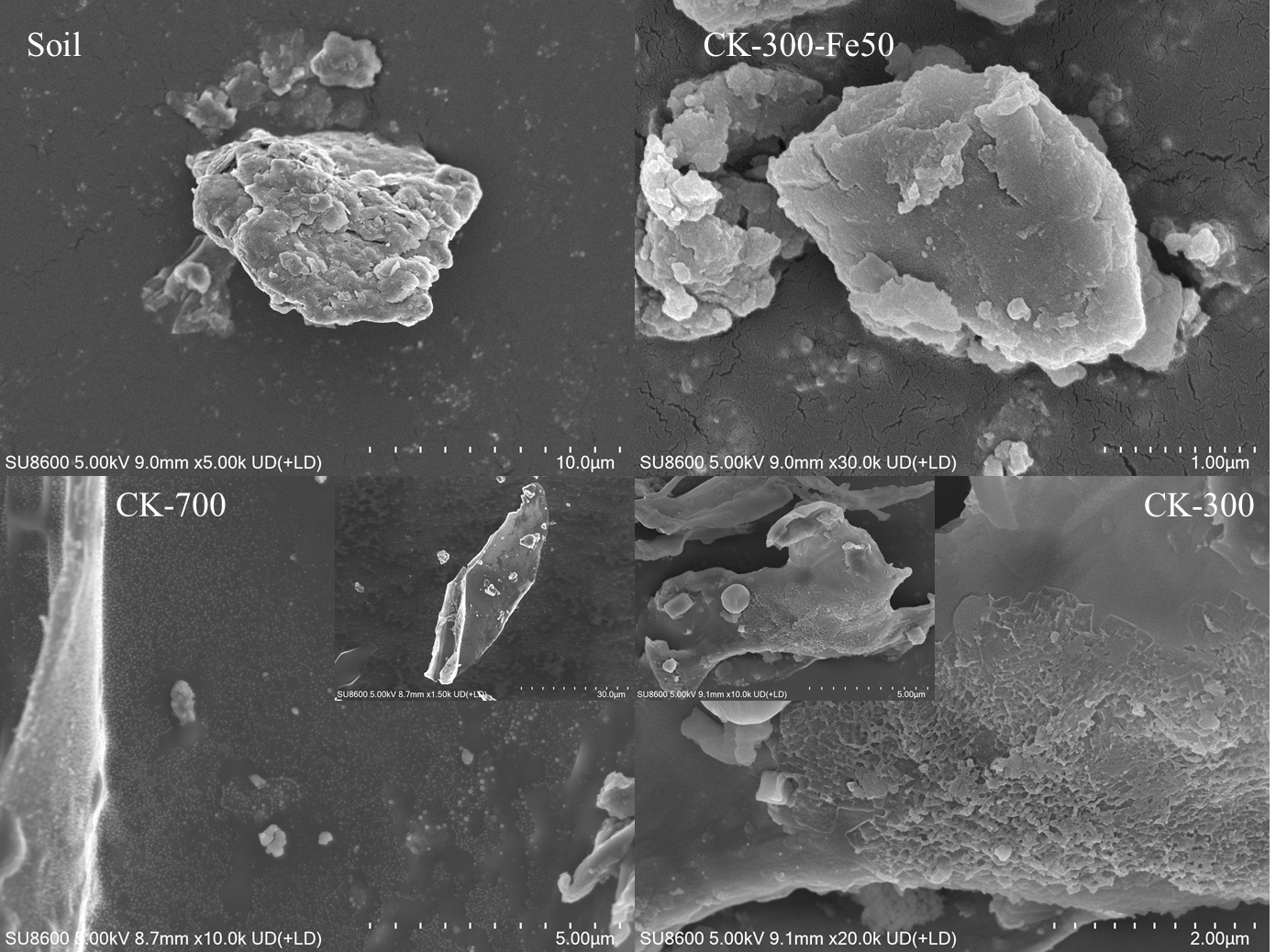


Fig. S1 SEM-EDX analysis of soil and different biochar composite materials

Fig. S2 XRD patterns of soil and different biochar composites

Fig. S3 FTIR spectra of soil and different biochar composites

Fig. S4 Removal kinetic curve of nitrobenzene (a and b) and aniline concentration (c) after reaction by different modified biochar composites

Fig. S5 Desorption concentration of nitrobenzene from soil

Fig. S6 TGA curves of wheat straw and biochar materials

a: wheat straw; b:CK-300; c:CK-700; d:CK-700; e: CK-700-Fe50; f: HNO_3_-700-Fe50; g: HCl-700-Fe50; h: NaOH-700-Fe50

Fig. S7 Residual ratio of nitrobenzene in the soil and water phases by different samples

Control: Soil; BC1: CK-300-Fe50-1%; BC2: CK-700-Fe50-1%; BC3: CK-700-Fe50-3%; BC4: HNO_3_-700-Fe50-3%; BC5: HCl-700-Fe50-3%; BC6: NaOH-700-Fe50-3%; BC7: CK-700-1%; BC8: CK-700-3%; BC9: CK-300-1%


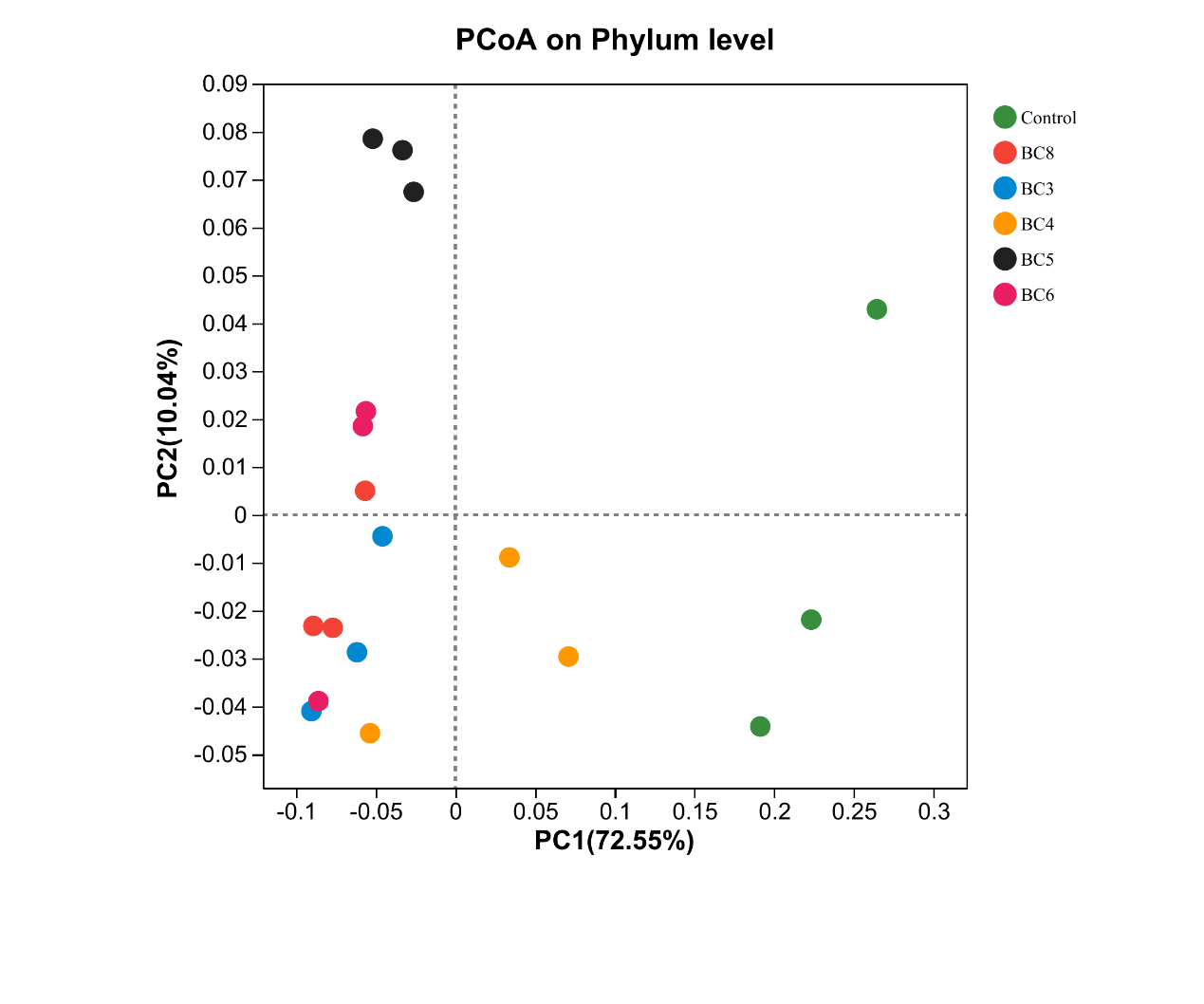


Fig. S8 Principal co-ordinates analysis (PCoA) of soil microbial communities in different treatments

Control: Soil; BC8:CK-700-3%; BC3:CK-700-Fe50-3%; BC4:HNO_3_-700-Fe50-3%; BC5:HCl-700-Fe50-3%; BC6:NaOH-700-Fe50-3%


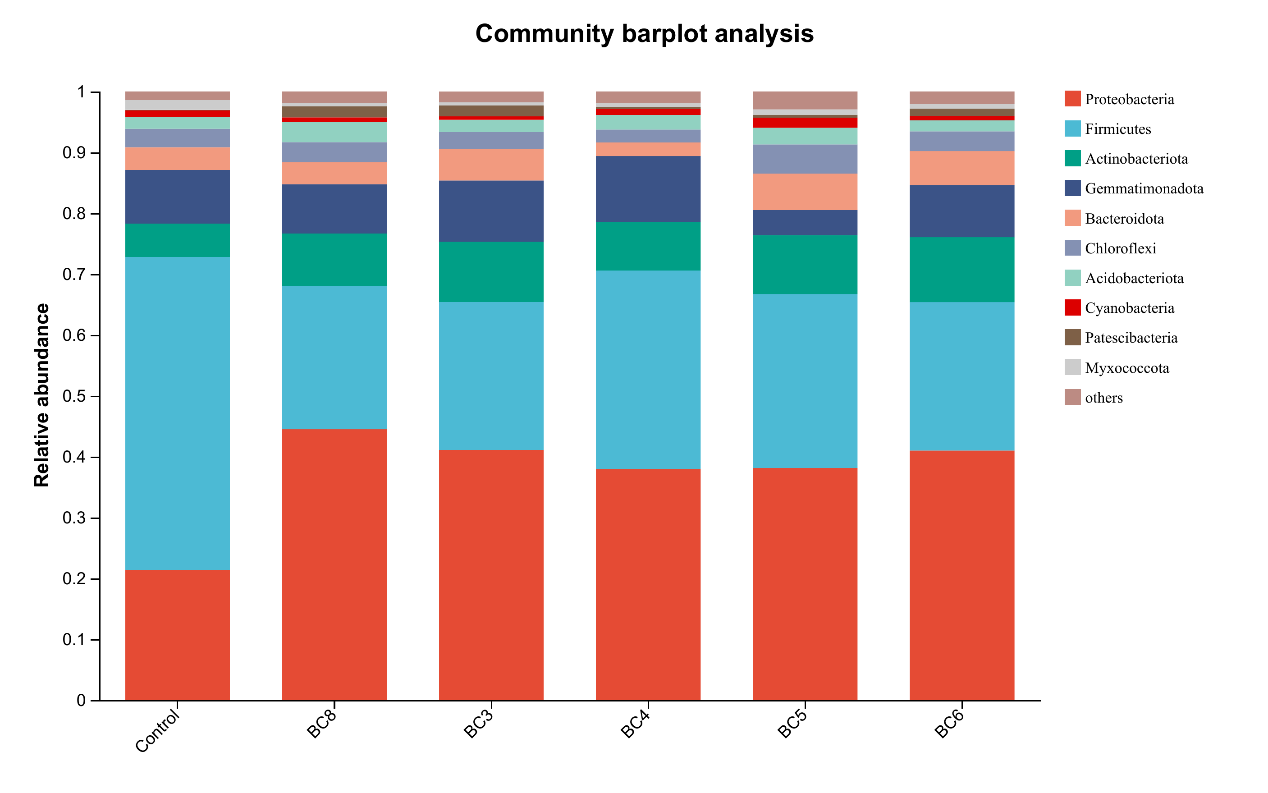


Fig. S9 Relative abundance of soil bacteria on phylum levels among different treatments

Control: Soil; BC8:CK-700-3%; BC3:CK-700-Fe50-3%; BC4:HNO_3_-700-Fe50-3%; BC5:HCl-700-Fe50-3%; BC6:NaOH-700-Fe50-3%


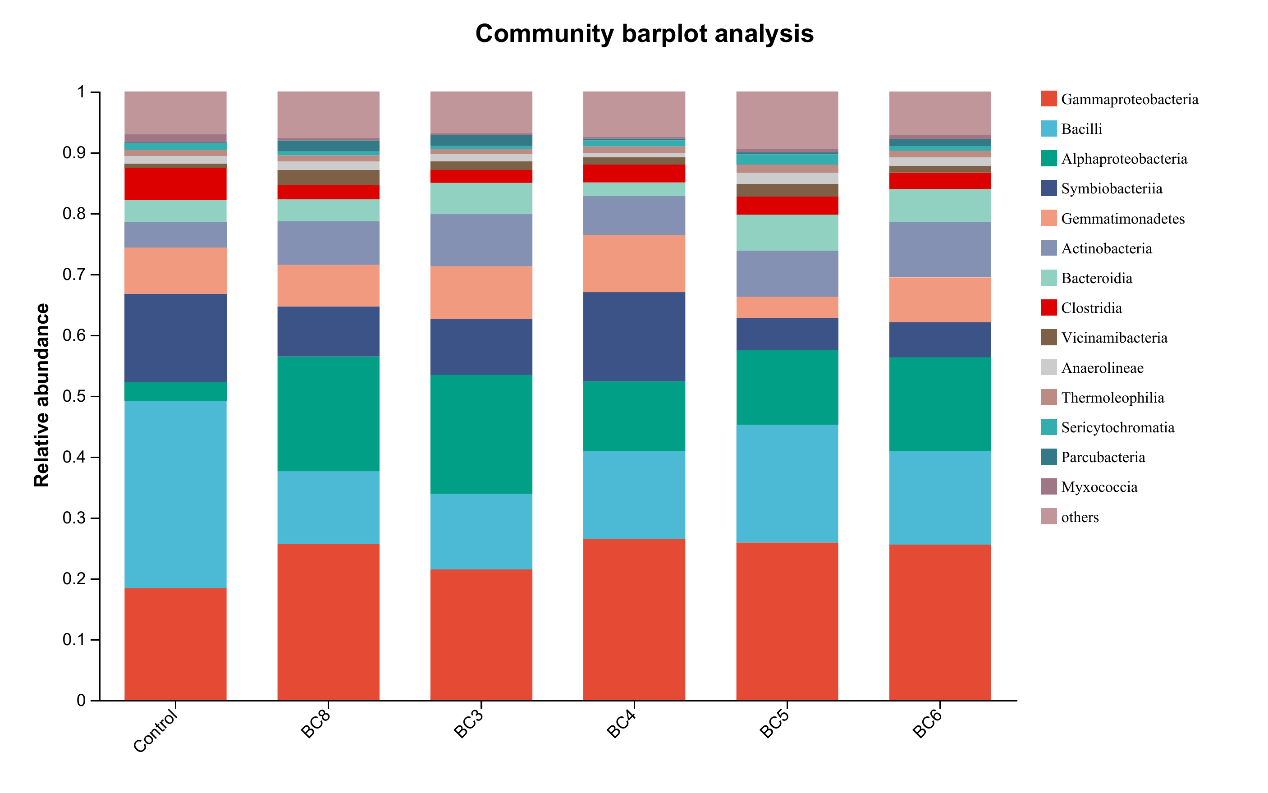


Fig. S10 The relative abundance of soil bacteria on class levels among different treatments

Control: Soil; BC8:CK-700-3%; BC3:CK-700-Fe50-3%; BC4:HNO_3_-700-Fe50-3%; BC5:HCl-700-Fe50-3%; BC6:NaOH-700-Fe50-3%


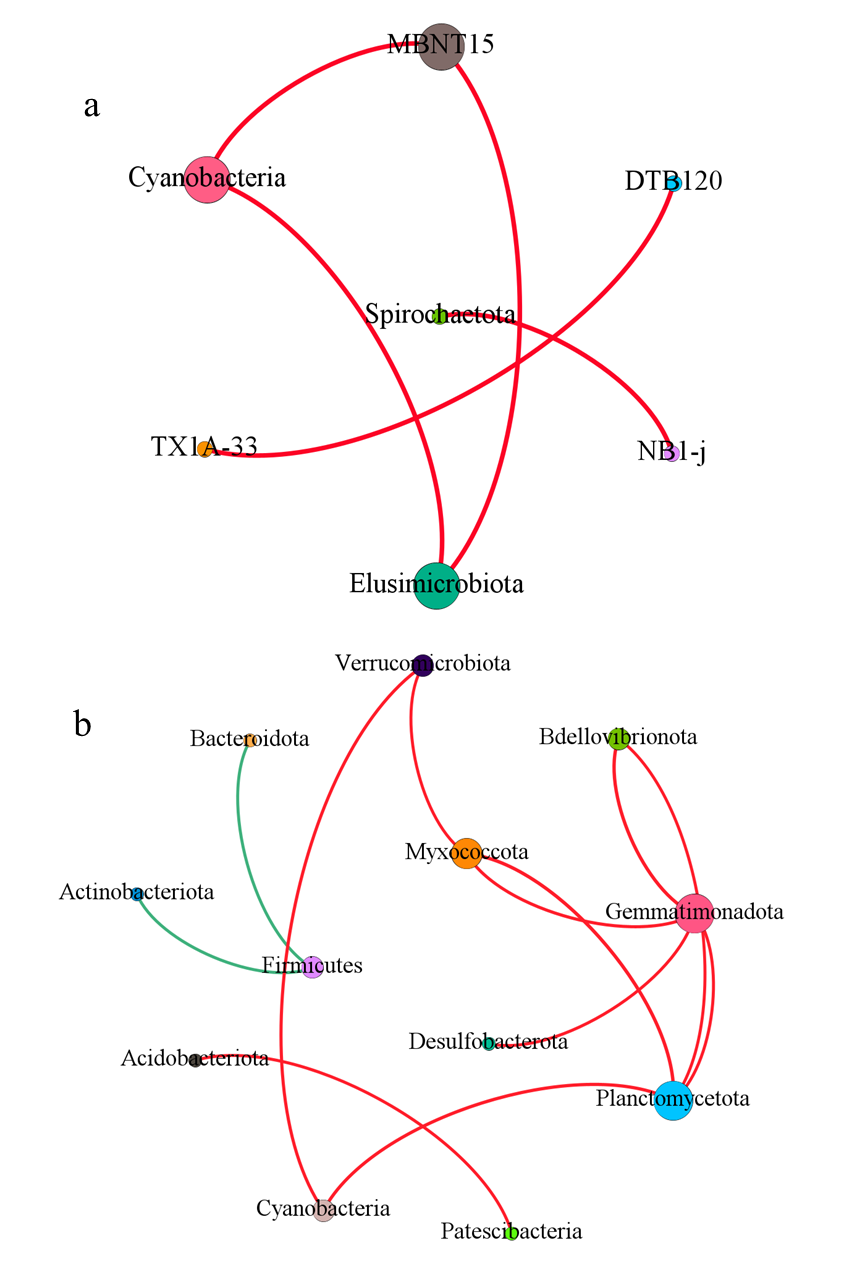


Fig. S11 The co-occurrence network of soil microbiota in non-ZVI addition samples (a) and ZVI addition samples (b) at phylum levels

Fig. S12 The potential related degradation genes in treatments.

Control: Soil; BC8:CK-700-3%; BC3:CK-700-Fe50-3%; BC4:HNO_3_-700-Fe50-3%; BC5:HCl-700-Fe50-3%; BC6:NaOH-700-Fe50-3%
